# Supplementary material for: Characterising the Response of Human Breast Cancer Cells to Polyamine Modulation
Source: Biomolecules. 2021 May 17;11(5):743. doi: 10.3390/biom11050743 (PMC8156773; doi:10.3390/biom11050743)
Supplement: Supplementary file 1 [file biomolecules-11-00743-s001.zip › biomolecules-1164696-supplementary.pdf]

Supplementary data

Supplementary data 1. Effect of exogenous polyamines on MDA-MB-232 cells growth

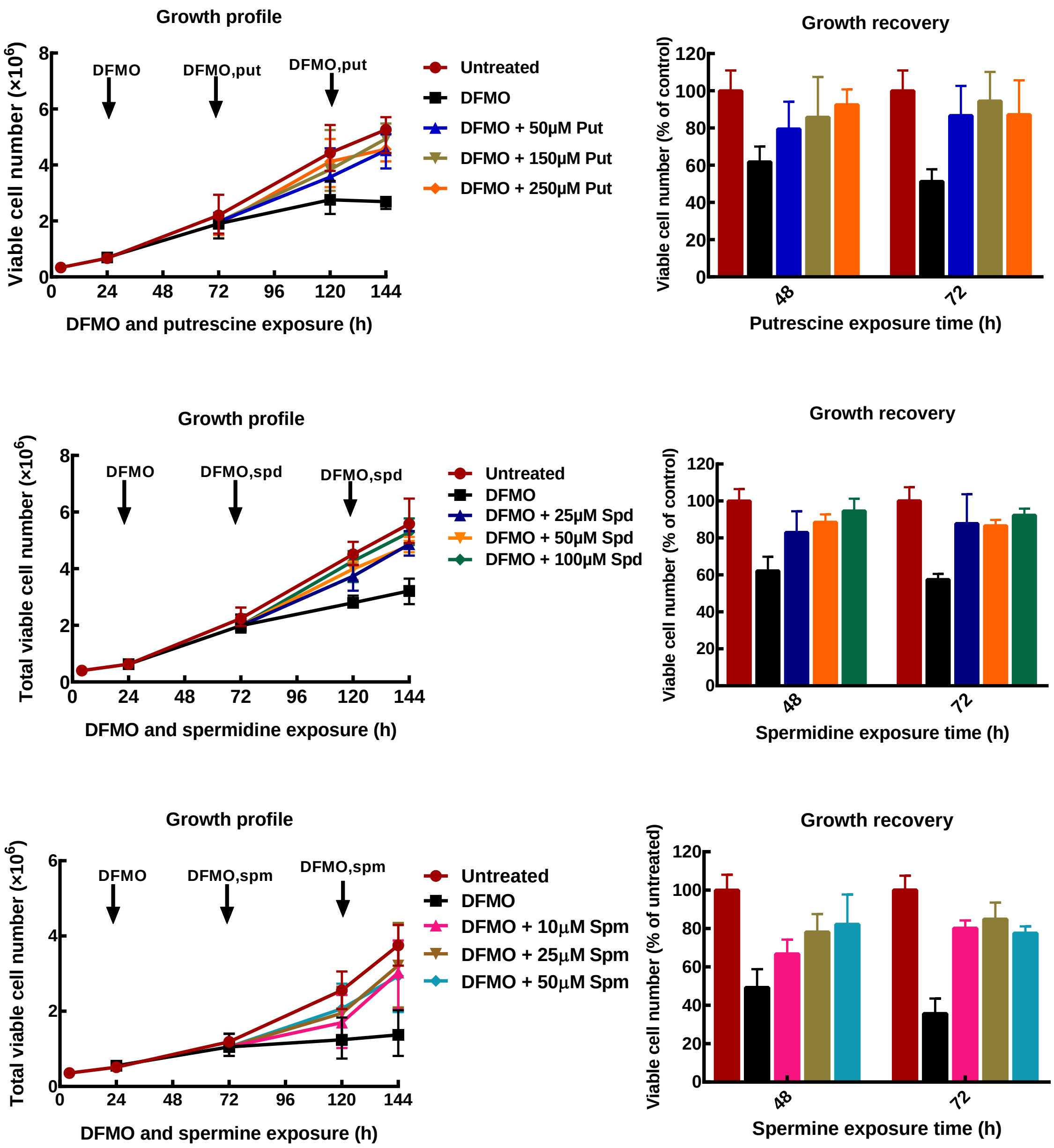

MDA-MB-231 cells were pre-treated with 5mM DFMO for 48 h, thereafter cells were treated with different concentrations of polyamines as indicated, following which cells number was determined. Cell growth profile and percentage growth recoveries by exogenous polyamines: (a) putrescine; (b) spermidine, and (c) spermine. Values shown are mean ± range (n=2) with 2 replicates per treatment.

Supplementary data 2. Intracellular polyamine content of MDA-MB-231 cells after exogenous polyamines additions

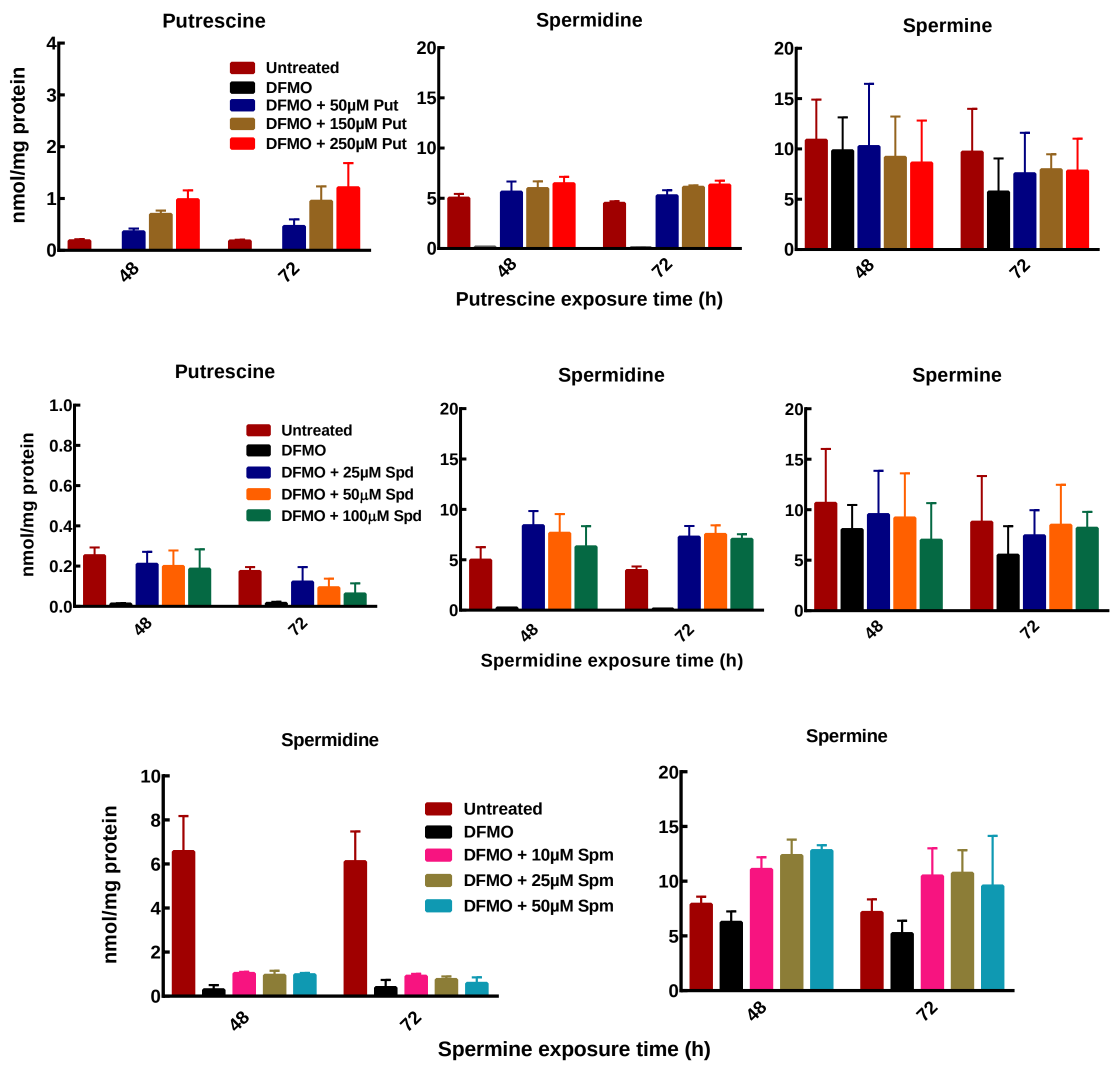

MDA-MB-231 cells were pre-treated with 5mM DFMO for 48 h, thereafter cells were treated with different concentrations of polyamines as indicated, following which cells were harvested and polyamines quantified. (a) polyamine content after exogenous putrescine addition, (b) polyamine content after exogenous spermidine addition, (c) polyamine content after exogenous spermine addition. Values shown are mean ± range (n=2) with 2 replicates per treatment.

Supplementary data 3. Effects of polyamine depletion on translation state of MDA-MB-231 cells

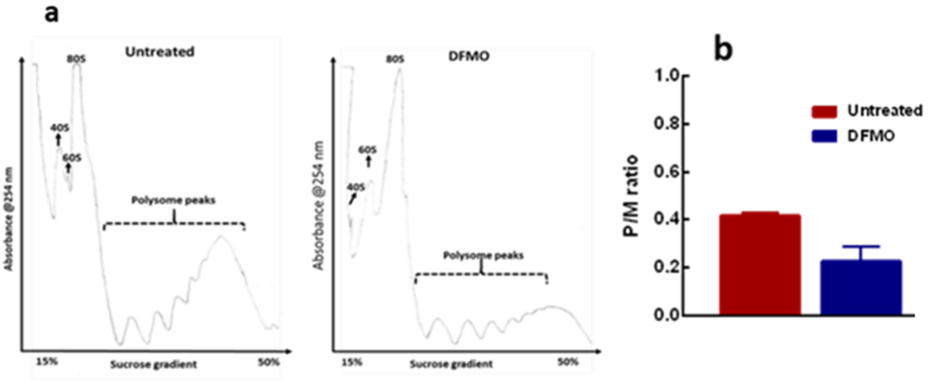

MDA-MB-231 cells were seeded, treated accordingly for 48 h, and harvested for polysome analysis. (a) Representative of polysome profiles from untreated control and 5 mM DFMO treatment. The images shown is a representative of two independent experiments. (b) Estimation of the polysome to monosome ratio for the respective treatment for area under curve, for and n=2.
